# The association of cardiometabolic disorders with sleep duration: a cross-sectional study

**DOI:** 10.4314/ahs.v22i4.32

**Published:** 2022-12

**Authors:** Dorcas R Prakaschandra, Datshanna P Naidoo

**Affiliations:** 1 Durban University of Technology; Department of Biomedical and Clinical Technology; 41/43 ML Sultan Road; Durban; 4000; South Africa; 2 University of KwaZulu-Natal; Department of Cardiology; Inkosi Albert Luthuli Central Hopsital; Bellair Road, Cator Manor; Durban; South Africa

**Keywords:** Sleep Time, Metabolic Syndrome X, Cardiovascular Risk

## Abstract

**Background:**

Alterations in sleep duration and quality are linked to the development of cardiovascular risk factors and the metabolic syndrome (MetS). The aim of this study was to determine a sex stratified analysis on the role and associations of sleep duration on cardiometabolic risk factors, and the MetS.

**Methods:**

Data from 1375 randomly selected participants (15–64 years) was collected for demographic, anthropometric, blood pressure and biochemistry data after overnight fasting, and derangements diagnosed according to published guidelines. Analysis of association between the MetS (harmonised criteria modified for South Asians), sleep duration (self-reported for a 24-hour period), and cardiometabolic risk factors was done using stepwise logistic regression.

**Results:**

The BMI, waist circumference (WC), systolic blood pressure (SBP) and diastolic blood pressure (DBP), fasting plasma glucose, total cholesterol, low density lipoprotein were higher (p< 0.05) in subjects who slept <6 hours, with lower HDL. Under 6 hours of sleep was independently associated with raised FPG in men (OR 1.71 95% CI [1.53,5.52]) only. More than 10 hours of sleep was independently associated with increased triglyceride levels in men (1.72[0.56, 5.23]) and women (2.25[1.93,5.42]).

**Conclusion:**

The individual components of the Mets, particularly, increased triglycerides and blood glucose are associated with sleep deprivation or excess.

## Introduction

Sleep duration and sleep quality are the two factors when assessing sleep health^1^, with the recent growing body of evidence indicating these factors are significantly associated with cardiovascular (CV) and metabolic risk factors^2^,^3^,^4^,^5^. A recent meta-analysis by Lian et al 6 links poor sleep quality with alterations in diurnal cortisol levels, increased markers of systemic inflammation and altered leptin levels and leptin resistance, all of which in turn have been linked to the development of insulin resistance and the metabolic syndrome (MetS)^6^. The MetS is an established risk factor for cardiovascular disease (CVD) as it is characterised by cardiovascular risk factors clustering^7^ and it is therefore reasonable to hypothesise that sleep duration and quality may also have associations with MetS. The rationale for this study stems from the inconclusive data on sleep duration and health outcomes, as well as for the associations in women^3^. In addition, there are no studies, which have been conducted on Asian Indians in this area. In view of the high prevalence of cardiovascular risk factors and the MetS which have been reported^8^, the aim of this study was to determine a sex-stratified analysis on the role and associations of sleep duration on cardiometabolic risk factors, as well as on the MetS.

## Methods

### Study population

Data from 1375 participants from the Phoenix Lifestyle project were studied, of whom 386 (28.1%) were men, and 989 (71.9%) women (Table 1). The detailed study design, research methodology and data collection has been published previously^8^. Briefly, this cross-sectional study studied data from 1375 randomly selected participants aged 15–64 years of the Phoenix community, in KwaZulu Natal using the Kish method of sampling 9.

**Table 1:** Baseline demographic, physiological and biochemistry of all participants

	Male (n=386; 28.1%)	Female (n=989; 71.9%)	Overall (n=1375)	p-value
**Age**	43.1±14.5	45.5±11.8	44.8±12.7	0.046
**Education**				
Less than primary	24 (6.2%)	180 (18.2%)	204 (14.8%)	< 0.001
Primary	94 (24.4%)	322 (32.6%)	416 (30.2%)	
Secondary	106 (27.5%)	240 (24.3%)	346 (25.1%)	
High school	121 (31.3%)	197 (19.9%)	321 (23.3%)	
University	15 (3.9%)	24 (2.4%)	39 (2.8%)	
Postgraduate	4 (1.0%)	1 (0.1%)	5 (0.4%)	
**Smoking status**	204 (52.8%)	139 (14.1%)	344 (25.0%)	< 0.001
**Alcohol**	188 (48.7%)	60 (6.1%)	249 (18.1%)	< 0.001
**BMI, kg/m^2^**	24.4±4.98(20.4)	29.0±6.33(21.8)	27.8±6.33(22.8)	0.005
**Waist, cm**	88.5±13.7(15.5)	95.1±15.2(16.0)	93.3±15.1(16.2)	< 0.001
**Neck, cm**	39.3±9.76(24.8)	35.9±9.27(25.8)	36.8±9.52(25.9)	< 0.001
**Systolic BP,** **mmHg**	133±19.4(14.6)	133±20.7(15.6)	133±20.3(15.3)	0.674
**Diastolic BP,** **mmHg**	80.5±13.0(16.1)	81.8±12.0(14.6)	81.4±12.3(15.1)	0.041
**FPG, mmol/L**	5.99±2.39(40.0)	6.41±2.95(46.0)	6.30±2.81(44.7)	0.003
**TC, mmol/L**	5.38±1.18(22.0)	5.46±1.17(21.4)	5.44±1.17(21.5)	0.105
**TG, mmol/L**	1.84±1.12(60.8)	1.72±1.04(60.7)	1.75±1.06(60.8)	<0.001
**HDL-C,** **mmol/L**	1.22±0.476(38.9)	1.35±0.440(32.7)	1.31±0.453(34.6)	0.679
**LDL-C, mmol/L**	3.34±1.10(32.8)	3.35±1.00(29.8)	3.35±1.03(30.7)	0.303
**Hypertension**	122 (31.6%)	317 (32.1%)	440 (31.9%)	0.812
**Diabetes**	96 (24.9%)	344 (34.8%)	441 (32.0%)	<0.001
**BMI**				<0.001
Normal	214 (55.4%)	265 (26.8%)	480 (34.8%)	
Overweight	124 (32.1%)	327 (33.1%)	451 (32.7%)	
Obese	48 (12.4%)	397 (40.1%)	447 (32.4%)	
**BMI (Asian cat)**				<0.001
Normal	187 (48.4%)	211 (21.3%)	399 (29.0%)	
Overweight	199 (51.6%)	778 (78.7%)	979 (71.0%)	
**MetS**	134 (34.7%)	496 (50.2%)	631 (45.8%)	<0.001
**Activity levels** **(METS)**				<0.001
Low	215 (55.7%)	465 (47.0%)	682 (49.5%)	
Medium	66 (17.1%)	292 (29.5%)	358 (26.0%)	
High	105 (27.2%)	232 (23.5%)	338 (24.5%)	
**Sleep duration**	7.03±1.68(23.9)	6.72±1.66(24.7)	6.80±1.67(24.5)	0.009
**Sleep duration** **categories**				< 0.001
<6hrs	61 (15.8%)	191 (19.3%)	252 (18.3%)	
6-<8hrs	154 (39.9%)	431 (43.6%)	588 (42.7%)	
8-<10hrs	146 (37.8%)	325 (32.9%)	471 (34.2%)	
10+hrs	20 (5.2%)	36 (3.6%)	56 (4.1%)	

Waist: waist circumference; Neck: neck circumference; FPG: fasting plasma glucose; TC: total cholesterol; HDL-C: high density lipoprotein; LDL-C: low density lipoprotein, BP: blood pressure

### Participant characteristics

The WHO STEPS modified questionnaire was used to collect demographic and anthropometric, as well as blood pressure and biochemistry data after overnight fasting. Participants with missing data for any of the parameters studied were excluded from this study.

Other data on parameters related to sleep quality and metabolic syndrome were also collected, for example, age, sex, education smoking and alcohol consumption status. Classification of metabolic and physiological derangements were as follows:

Hypertension was defined in individuals who self-reported previously diagnosed hypertension, and/or those with blood pressure readings ≥140 and ≥90 mmHg (average of three readings) (Joint National Committee VII (JNC VII) criteria) and/or those on current antihypertensive therapy^10^.

Diabetes was defined in individuals who self-reported and who were currently on medication. In all others, the WHO criteria^11^ were used to classify glycaemic categories based on fasting plasma glucose (FPG).

The MetS was defined using the harmonised criteria^7^ when any three of the following five criteria were present, modified for South Asians: increased waist circumference (>90 cm [men]; >80 cm[women]), elevated triglycerides (>1.7 mmol/L); reduced HDL cholesterol (<1.03 mmol/L [<1.29 mmol/L [women]); elevated blood pressure 130 and/85 mmHg and increased fasting plasma glucose (>5.6 mmol/L).

### Assessment of sleep quality

The STEPS questionnaire instrument consists of three items related to sleep quality in the form of the following questions:

- Have you or anyone noticed that you quit breathing during your sleep?
- Overall, in the last 30 days, how much of a problem did you have with sleeping, such as falling asleep, waking up frequently during the night or waking up too early in the morning?
- In the last 30 days, how much of a problem did you have due to not feeling rested and refreshed during the day (e.g., feeling tired, not having energy)?

### Assessment of sleep duration

For the PLP study, sleep duration was self-reported and assessed using the question: “How many hours do you sleep during a 24-hour period?” The responses were then converted into four sleep category responses as used by Yoon et al^12^. In the multivariable analysis, 6 to < 8 h category was selected as reference since 6 to < 8 h is the median sleep category that was observed in this sample.

### Statistical analysis

Statistical analysis was performed using R 3.3.1 statistical computing language (R Core Team, 2016). Baseline and demographic data are presented descriptively using mean +/- SD. The Kruskal-Wallis H Test (for categorical variables) and ANOVA (for continuous variables) were used to compare the demographic and clinical characteristics of participants between the four categories of sleep duration, and differences between men and women. A multivariable stepwise analysis was performed to calculate the odds ratios (ORs) and 95% confidence intervals (95% CIs), after adjustment for potential confounding parameters like age, sex, smoking, alcohol consumption, sleeping problems and physical activity. All parameters were adjusted for each other in the final model presented. Ethical approval was granted by the University of Kwa-Zulu-Natal Bioethics committee (Ethics reference: E336/05) and conformed to the principles in the Declaration of Helsinki. Informed consent was acquired from each participant before the collection of this data, and all were informed of the results of the examinations undertaken. Subjects in whom risk factors were identified were referred to a health facility for further evaluation and management.

## Results

This study examined the data of 1375 participants, predominantly women (989; 71.9%) and 386 men (28.1%), mean age of the sample being 44.8±12.7 years (Table 1). The demographic features of the sample, stratified according to men and women are shown in Table 1.

Although there were similar proportion of men and women attending primary and high school, there were significantly fewer attending university and beyond (p<0.001). There were significantly higher numbers of men who smoked and consumed alcohol, and had a higher neck circumference, when compared to women (p<0.001). This pattern was reversed for the BMI and waist circumference, where these were significantly higher in women than in men.

Biochemical analysis showed a higher fasting plasma glucose in women and a higher triglyceride level in men. A significantly higher proportion of women were obese and classified with diabetes and with the MetS (p<0.001). A significantly higher number of individuals engaged in low activity levels. The mean duration of sleep was 6.80±1.67 hours and was significantly lower in women (p = 0.009). When demographics were classified according to sleep duration category (Table 2), consumers of alcohol (32.1%) were more inclined to sleep for longer than 10 hours. A higher proportion of participants who complained that they quit breathing in their sleep, those with severe sleep problems (63.5%) and those who awoke extremely non-refreshed (52.8%) slept for < 6 hours per night.

**Table 2:** Demographic, physiological and biochemistry of all participants stratified by sleep duration

	<6hrs (n=252)	6? 8hrs (n=588)	8? 10hrs (n=471)	>10hrs (n=56)	Overall (n=1378)	P- value
**Sex**						0.095
Male	61 (24.2%)	154 (26.2%)	146 (31.0%)	20 (35.7%)	386 (28.0%)	
Female	191 (75.8%)	431 (73.3%)	325 (69.0%)	36 (64.3%)	989 (71.8%)	
Age	46.8±11.5(24.5)	45.5±11.8(25.9)	43.8±13.5(30.8)	38.0±16.3(42.8)	44.8±12.7(28.3)	0.001
**Educational** **level**						
Less than primary	50 (19.8%)	81 (13.8%)	67 (14.2%)	6 (10.7%)	204 (14.8%)	0.245
Primary	78 (31.0%)	177 (30.1%)	145 (30.8%)	15 (26.8%)	416 (30.2%)	
Secondary	65 (25.8%)	149 (25.3%)	121 (25.7%)	10 (17.9%)	346 (25.1%)	
High school	47 (18.7%)	143 (24.3%)	111 (23.6%)	19 (33.9%)	321 (23.3%)	
University	4 (1.6%)	18 (3.1%)	13 (2.8%)	4 (7.1%)	39 (2.8%)	
Postgraduate	1 (0.4%)	3 (0.5%)	1 (0.2%)	0 (0%)	5 (0.4%)	
**Smokers**	69 (27.4%)	142 (24.1%)	115 (24.4%)	16 (28.6%)	344 (25.0%)	0.687
Alcohol	39 (15.5%)	106 (18.0%)	86 (18.3%)	18 (32.1%)	249 (18.1%)	0.029
**Quit** **breathing**	43 (17.1%)	67 (11.4%)	45 (9.6%)	9 (16.1%)	164 (11.9%)	0.022
**Sleep** **problems**						
None	4 (1.6%)	66 (11.2%)	69 (14.6%)	13 (23.2%)	152 (11.0%)	<0.001
Mild	15 (6.0%)	81 (13.8%)	116 (24.6%)	13 (23.2%)	226 (16.4%)	
Moderate	25 (9.9%)	226 (38.4%)	169 (35.9%)	20 (35.7%)	441 (32.0%)	
Severe	48 (19.0%)	77 (13.1%)	48 (10.2%)	6 (10.7%)	180 (13.1%)	
Extreme	160 (63.5%)	134 (22.8%)	67 (14.2%)	4 (7.1%)	365 (26.5%)	
**Non-** **refreshed**						
None	10 (4.0%)	55 (9.4%)	48 (10.2%)	10 (17.9%)	123 (8.9%)	<0.001
Mild	10 (4.0%)	76 (12.9%)	104 (22.1%)	9 (16.1%)	199 (14.4%)	
Moderate	56 (22.2%)	244 (41.5%)	202 (42.9%)	18 (32.1%)	522 (37.9%)	
Severe	43 (17.1%)	96 (16.3%)	54 (11.5%)	11 (19.6%)	205 (14.9%)	
Extreme	133 (52.8%)	116 (19.7%)	61 (13.0%)	8 (14.3%)	318 (23.1%)	
**BMI,** **kg/m^2^**	28.3±6.51(23.0)	27.9±5.86(21.0)	27.6±6.65(24.1)	25.0±6.73(26.9)	27.8±6.33(22.8)	0.005
**Waist, cm**	94.4±14.1(15.0)	94.0±14.2(15.2)	92.7±16.3(17.6)	86.0±15.1(17.5)	93.3±15.1(16.2)	<0.001
**Neck, cm**	36.9±9.74(26.3)	36.5±8.82(24.2)	36.9±9.54(25.9)	40.0±14.6(36.4)	36.8±9.52(25.9)	0.792
**FPG,** **mmol/L**	6.63±2.87(43.3)	6.28±2.78(44.3)	6.27±2.82(45.0)	5.46±2.87(52.6)	6.30±2.81(44.7)	<0.001
**TC,** **mmol/L**	5.67±1.26(22.1)	5.41±1.10(20.3)	5.38±1.20(22.2)	5.20±1.17(22.4)	5.44±1.17(21.5)	0.011
**TG,** **mmol/L**	1.81±0.905(50.1)	1.73±1.03(59.3)	1.73±1.14(66.0)	1.87±1.42(76.0)	1.75±1.06(60.8)	0.101
**HDL-C,** **mmol/L**	1.30±0.510(39.3)	1.31±0.495(37.9)	1.31±0.344(26.2)	1.45±0.535(37.0)	1.31±0.453(34.6)	0.081
**LDL-C,** **mmol/L**	3.55±1.18(33.3)	3.35±0.962(28.7)	3.27±0.992(30.3)	3.02±1.11(36.7)	3.35±1.03(30.7)	0.004
**Systolic BP,** **mmHg**	134±21.1(15.7)	133±19.8(14.9)	134±20.5(15.4)	126±20.0(15.9)	133±20.3(15.3)	0.01
**Diastolic** **BP, mmHg**	81.8±11.4(13.9)	81.6±12.5(15.3)	81.4±12.2(15.0)	77.7±13.2(17.0)	81.4±12.3(15.1)	0.067
**BMI**						
Normal	87 (34.5%)	196 (33.3%)	166 (35.2%)	27 (48.2%)	480 (34.8%)	0.448
Overweight	83 (32.9%)	194 (33.0%)	152 (32.3%)	17 (30.4%)	451 (32.7%)	
Obese	82 (32.5%)	198 (33.7%)	153 (32.5%)	12 (21.4%)	447 (32.4%)	
**BMI** **(Asian)**						
Normal	61 (24.2%)	156 (26.5%)	151 (32.1%)	27 (48.2%)	399 (29.0%)	<0.001
Overweight	191 (75.8%)	432 (73.5%)	320 (67.9%)	29 (51.8%)	979 (71.0%)	
**Diabetes**	96 (38.1%)	193 (32.8%)	143 (30.4%)	8 (14.3%)	441 (32.0%)	0.006
**Physical** **activity** **(METS)**						
Low	107 (42.5%)	291 (49.5%)	238 (50.5%)	37 (66.1%)	682 (49.5%)	0.01
Medium	69 (27.4%)	144 (24.5%)	134 (28.5%)	9 (16.1%)	358 (26.0%)	
High	76 (30.2%)	153 (26.0%)	99 (21.0%)	10 (17.9%)	338 (24.5%)	

Waist: waist circumference; Neck: neck circumference; FPG: fasting plasma glucose; TC: total cholesterol; HDL-C: high density lipoprotein; LDL-C: low density lipoprotein, BP: blood pressure

The BMI, waist circumference (WC), systolic blood pressure (SBP) and diastolic blood pressure (DBP) were higher (p< 0.05) in subjects who slept < 6 hours than those in the other groups. A similar pattern where higher fasting plasma glucose (FPG), total cholesterol (TC), low density lipoprotein (LDL) and lower HDL was observed in this group. Triglyceride (TG) levels was the highest in subjects who slept >10 hours. These findings were more prominent in females than in males.

The overall prevalence of the MetS was 45.8% and was significantly higher in women (50.2%) than in men (34.7%).

The highest number of participants with MetS were seen in the 6 - <8 hours group (n=278). There were significant statistical differences (p< 0.05) for the following MetS co-variates: blood pressure, waist circumference and FPG among the sleep duration categories, with the highest proportion of individuals with metabolic derangements in those who slept for less than 6 hours (Table 3).

**Table 3:** Analysis of the metabolic syndrome (MetS) and its components prevalent by sleep duration

	<6hrs (n=252)	6≤8hrs (n=588)	8≤10hrs (n=471)	>10hrs (n=56)	Overall (n=1378)	p-value
**Metabolic** **syndrome**	131 (52.0%)	278 (47.3%)	204 (43.3%)	14 (25.0%)	631 (45.8%)	0.002
**TG, mmol/L**	114 (45.2%)	238 (40.5%)	192 (40.8%)	23 (41.1%)	570 (41.4%)	0.625
**HDL-C**	115 (45.6%)	258 (43.9%)	194 (41.2%)	24 (42.9%)	597 (43.3%)	0.666
**BP ≥130/85** **mmHg**	86 (34.1%)	180 (30.6%)	165 (35.0%)	8 (14.3%)	440 (31.9%)	0.011
**WC, cm**	196 (77.8%)	447 (76.0%)	333 (70.7%)	25 (44.6%)	1007 (73.1%)	<0.001
**FPG,** **mmol/L**	107 (42.5%)	229 (38.9%)	166 (35.2%)	11 (19.6%)	517 (37.5%)	0.008

The MetS: any 3 of the following, modified for South Asians: WC: increased waist circumference (>90 cm [men]; >80 cm[women]); TG: triglycerides (>1.7 mmol/L); HDL-C: reduced HDL cholesterol (<1.03 mmol/L(men) [<1.29 mmol/L [women]); BP: elevated blood pressure 130 and/85 mmHg; FPG: increased fasting plasma glucose (>5.6 mmol/L).

The ORs and 95% CIs for the metabolic syndrome according to sleep duration categories were calculated using multiple logistic regression analyses (Table 4).

**Table 4:** Logistic regression analysis of the MetS by sleep duration

	<6hrs	6≤8hrs	8≤<10hrs	>10hrs
All	(n=252)	(n=588)	(n=471)	(n=56)
MetS	1.02(0.62,1.68)	reference	0.81(0.53,1.23)	0.53(0.2,1.43)
WC, cm	0.97(0.65,1.44)	reference	0.77(0.57,1.06)	0.33(0.17,0.63)
FPG, mmol/L	1.11(0.78,1.58)	reference	0.9(0.67,1.21)	0.61(0.28,1.33)
TG, mmol/L	1.18(0.81,1.71)	reference	1.19(0.87,1.63)	1.95(1.99,3.84)
HDL-C, mmol/L	1(0.71,1.41)	reference	0.98(0.74,1.31)	1.39(0.74,2.62)
BP≥130/≥85	1.12(0.79,1.58)	reference	1.39(1.04,1.85)	0.6(0.26,1.38)
**Men**	**n= 61**	**n=154**	**n=146**	**n=20**
Age	0.99(0.31,3.15)	reference	0.99(0.96,1.01)	1.01(0.97,1.05)
MetS	0.63(0.21,1.91)	reference	0.98(0.42,2.26)	0.33(0.04,2.56)
WC, cm	1.1(0.92,1.31)	reference	0.92(0.5,1.67)	0.75(0.21,2.62)
FPG, mmol/L	1.71(1.53,5.52)	reference	1.03(0.58,1.81)	0.47(0.09,2.38)
TG, mmol/L	1.07(0.5,2.29)	reference	1.43(0.8,2.53)	1.72(1.56,5.23)
HDL-C, mmol/L	0.66(0.3,1.43)	reference	0.94(0.54,1.63)	0.93(0.28,3.06)
BP≥130/≥85	0.85(0.4,1.77)	reference	1.3(0.75,2.25)	0.44(0.09,2.17)
**Women**	**n=191**	**n=431**	**n=325**	**n=36**
Age	0.99(0.97,1.02)	reference	1.01(0.99,1.03)	0.95(0.91,0.98)
MetS	1.11(0.62,1.96)	reference	0.79(0.48,1.29)	0.49(0.15,1.65)
WC, cm	0.88(0.51,1.52)	reference	0.66(0.43,1.01)	0.23(0.1,0.51)
FPG, mmol/L	1.05(0.7,1.58)	reference	0.86(0.6,1.22)	0.7(0.28,1.72)
TG, mmol/L	1.24(0.81,1.91)	reference	1.06(0.72,1.54)	2.25(1.93,5.42)
HDL-C, mmol/L	1.1(0.74,1.62)	reference	0.99(0.71,1.39)	1.65(0.76,3.6)
BP≥130/≥85	1.21(0.81,1.79)	reference	1.37(0.97,1.93)	0.68(0.25,1.8)

MetS: metabolic syndrome; Waist: waist circumference; Neck: neck circumference; FPG: fasting plasma glucose; TC: total cholesterol; HDL-C: high density lipoprotein; LDL-C: low density lipoprotein, BP: blood pressure

After adjustment for potential confounders (age, gender, educational level, smoking and alcohol consumption, sleeping problems, and physical activity), sleep duration was not an independent predictor of the MetS. However, in terms of individual components of the MetS, the OR of increased TG almost doubled (OR 95% CI [1.95(1.99,3.84] in participants who slept for >10 hours. Less than 6 hours of sleep was independently associated with raised FPG in men (OR 1.71 95% CI [1.53,5.52]), but not in women (OR 1.05 95% CI [0.7,1.58]). More than 10 hours of sleep was independently associated with increased triglyceride levels in both men (1.72[0.56, 5.23]) and women (2.25[1.93,5.42]). Sleep duration was not a predictor of the MetS in either men or women.

## Discussion

There is a high prevalence of the MetS in Asian Indians in South Africa, and as such, it has become crucial to identify the modifiable risk factors associated with the MetS and its components. To the best of our knowledge, this is the first such study which provides information on sleep duration and sleep complaints like quitting breathing while sleeping, problems sleeping and awaking non-refreshed in a community sample of Asian Indians in South Africa. In addition, this is the first that has sought to determine the association between sleep duration and quality and cardiometabolic risk factors, as well as the MetS in men and women in this ethnic group in South Africa. The main findings were that sleep duration was not associated with the MetS, but sleep duration in terms of deprivation and excess, conferred increased risk for individual MetS components.

Chronic sleep loss (< 6 hours) or deprivation, was present in a significantly higher proportion of participants (52.0%) with the MetS when compared to the other duration groups, as well as those with increased waist circumference and increased FPG (Table 2). This is consistent with other studies which link sleep deprivation with such conditions as diabetes, hypertension^13^, depression^14^, obesity and even increased mortality^5^. The underlying mechanisms for our findings with regards to the increased waist circumference and FPG in participants who slept for less than 6 hours may be attributed to the increased ghrelin and leptin levels, which in turn increases hunger, decreases satiety, tipping the energy balance to excess levels^15^. This mechanism has also been reported to contribute to poorer glycaemic control and may account for the fasting glucose derangements seen in both men and women in our study, as well as the highest proportion of diabetic individuals (38.1%) who slept for less than 6 hours per night (Table 2). A pro-inflammatory state induced by sleep deprivation is another possible explanation for poor glycaemic control, as inflammatory biomarkers have also been linked to the development of cardiovascular disease^16^. In fact, a recent paper by Hijmans et al^17^ showed that dysregulation of circulating levels of miR-125a, miR-126 and miR-146a, which was observed in chronic short sleep, was associated with an increased inflammatory burden and endothelial dysfunction. These miRs have also been shown to amply the inflammatory burden in the obese^18^. Bromley & Booth^19^ found that sleep restriction was also associated with negative behavioural modification in the form of an increase in sedentary behaviour and may explain the link between sleep deprivation and increased BMI and waist circumference in this group.

Our findings confirm that the risk associated with changes in sleep duration is influenced by gender^20^, as men who slept < 6 hours had a 71% higher likelihood of increased fasting plasma glucose; this was not the case in women (Table 1). However, both men and women who slept for > 10 hours had an increased propensity for higher fasting triglyceride levels, similar to the findings by Smiley et al^21^. The possible mechanisms remain unclear for the association between long sleep duration and increased triglyceride levels. However, it is postulated that confounding and co-morbidities like low socio-economic status, low levels of physical activity and undiagnosed health conditions may have a role in this^22^.

The literature remains divided on the association between sleep duration and MetS; several studies^21^,^23^ found a U-shaped association between short and long sleep duration and MetS, whereas other recent studies^24^,^25^ did not. As such, our study did not show any association between the MetS and sleep duration or quality (Table 3). In addition, although lifestyle related to sleep disturbances have been postulated to underpin the mechanisms of the association between sleep duration or quality and MetS 26, we were not able to show a significant relationship for physical activity, smoking or alcohol consumption in our study. However, our study supports previous studies (Table 4) where individual components of the MetS was associated with sleep deprivation or excess^20^,^27^. These discrepancies may be explained by the variability of these individual components in the different population groups with the MetS^7^, as well due to disparities in socio-economic factors, ethnicity and diagnostic criteria for MetS^28^.

## Conclusion

This study confirms that individual components of the Mets, particularly, increased triglycerides and blood glucose are associated with sleep deprivation or excess. The value of our study lies in that it points to possible associations, but do not establish a cause-and-effect relationship; therefore, additional studies should be performed in order to clarify the underlying relationship between the sleep duration and the development of these pathophysiologies. This study highlights the importance of sleep in the clinical management CVD and therefore recommends that individuals with either a short or long sleep duration should be evaluated for glucose and triglyceride derangements.

## Limitations

The cross-sectional nature of this study limits causal relationships between sleep duration and metabolic abnormalities, since it was assessed at one point in time. It is therefore possible that this single measure may not fully reflect the continued effects of sleep duration over time. In addition, reverse causality must also be considered to contribute to sleep deprivation, since we could not account for the effects of pain and polyuria that may disrupt sleep on participants since these were not controlled for. Secondly, there is still debate around whether use of a questionnaire for measuring disturbed sleep is a reliable and valid method; also, the sleep duration was self-reported, and thus the reliability of these measures could not be substantiated by objective sleep measured like actigraphy, polysomnography or sleep journals. Nonetheless, these methods are the most frequently used for estimating sleep disturbances, particularly in epidemiological studies 20.

## What is already know on this topic

Sleep duration and sleep quality are the two factors when assessing sleep health, with recent evidence indicating that these factors are significantly associated with cardiovascular (CV) and metabolic risk factors.

There is inconclusive data for sleep duration and health outcomes, as well as the associations in women; additionally, there are no studies which have been conducted on Asian Indians, or in South Africa in this area.

## What this study adds

This study reports that individual components of the Mets, particularly, increased triglycerides and blood glucose are associated with sleep deprivation or excess and adds to the current body of knowledge in this understudied group.

This study highlights the importance of sleep in the clinical management CVD and therefore recommends that individuals with either a short or long sleep duration should be evaluated for glucose and triglyceride derangements.

## References

[1] Buysse DJ (2014). Sleep health: can we define it? Does it matter?. Sleep.

[2] Shayestefar M, Haghighi S, Jahanfar S, Delvarianzadeh M, Nematzadeh F, Ebrahimi MH (2019). Assessment of the relationship between metabolic syndrome and obstructive sleep apnea in male drivers of Shahroud city in 2018: A cross sectional study. BMC Public Health.

[3] Massar SAA, Liu JCJ, Mohammed NB, Chee MWL (2017). Poor habitual sleep efficiency is associated with increased cardiovascular and cortisol stress reactivity in men. Psychoneuroendocrinology.

[4] Tsai HJ, Kuo TB, Lin YC, Yang CC (2015). The association between prolonged sleep onset latency and heart rate dynamics among young sleep-onset insomniacs and good sleepers. Psychiatry Res.

[5] Cappuccio FP, D'Elia L, Strazzullo P, Miller MA, Sleep duration (2010). and all-cause mortality: a systematic review and meta-analysis of prospective studies. Sleep.

[6] Lian Y, Yuan Q, Wang G, Tang F (2019). Association between sleep quality and metabolic syndrome: A systematic review and meta-analysis. Psychiatry Research.

[7] Alberti KG, Eckel RH, Grundy SM, Zimmet PZ, Cleeman JI, Donato KA (2009). Harmonizing the metabolic syndrome: a joint interim statement of the International Diabetes Federation Task Force on Epidemiology and Prevention; National Heart, Lung, and Blood Institute; American Heart Association; World Heart Federation; International Atherosclerosis Society; and International Association for the Study of Obesity. Circulation.

[8] Prakaschandra R, Naidoo DP (2017). Increased waist circumference is the main driver for the development of the metabolic syndrome in South African Asian Indians. Diab Met Syndr: Clin Res Rev.

[9] Kish L (1995). Survey Sampling.

[10] Chobanian AV, Bakris GL, Black HR (2003). Seventh Report of the Joint National Committee on Prevention, Detection, Evaluation, and Treatment of High Blood Pressure. Hypertension.

[11] World Health Organization and International Diabetes Federation (2005). Definition and diagnosis of diabetes mellitus and intermediate hyperglycemia: Report of a WHO/IDF consultation.

[12] Yoon HS, Lee KM, Yang JJ, Lee HW, Song M, Lee SA, Lee JK, Kang D (2016). Associations of sleep duration with metabolic syndrome and its components in adult Koreans: from the health examinees study. Sleep Biol Rhythms.

[13] Wang Q, Xi B, Liu M, Zhang Y, Fu M (2012). Short sleep duration is associated with hypertension risk among adults: a systematic review and meta-analysis. Hypertens Res.

[14] Roberts RE, Duong HT (2014). The prospective association between sleep deprivation and depression among adolescents. Sleep.

[15] Taheri S, Lin L, Austin D, Young T, Mignot E (2004). Short sleep duration is associated with reduced leptin, elevated ghrelin, and increased body mass index. PLoS Med.

[16] Grandner MA, Sands-Lincoln MR, Pak VM, Garland SN (2013). Sleep duration, cardiovascular disease, and proinflammatory biomarkers. Nat Sci Sleep.

[17] Hijmans JG, Levy M, Garcia V, Lincenberg GM, Diehl KJ, Greiner JJ, Stauffer BL, DeSouza CA (2019). Insufficient sleep is associated with a pro-atherogenic circulating microRNA signature. Exp Physiol.

[18] Hijmans JG, Diehl KJ, Bammert TD, Kavlich PJ, Lincenberg GM, Greiner JJ, Stauffer BL, DeSouza CA (2018). Influence of Overweight and Obesity on Circulating Inflammation-Related microRNA. Microrna.

[19] Bromley LE, Booth JN, Kilkus JM, Imperial JG, Penev PD (2012). Sleep restriction decreases the physical activity of adults at risk for type 2 diabetes. Sleep.

[20] Leineweber C, Kecklund G, Åkerstedt T, Janszky I, Orth-Gomér K (2003). Snoring and the metabolic syndrome in women. Sleep Medicine.

[21] Smiley A, Wolter S, Nissan D (2019). Mechanisms of Association of Sleep and Metabolic Syndrome. J Med - Clin Res & Rev.

[22] Krueger PM, Friedman EM (2009). Sleep duration in the United States: a cross-sectional population-based study. Am J Epidemiol.

[23] Ju SY, Choi WS (2013). Sleep duration and metabolic syndrome in adult populations: a meta-analysis of observational studies. Nutr Diabetes.

[24] Wu SH, Liu Z, Ho SC (2010). Metabolic syndrome and all-cause mortality: a meta-analysis of prospective cohort studies. Eur J Epidemiol.

[25] Xi B, He D, Zhang M, Xue J, Zhou D (2014). Short sleep duration predicts risk of metabolic syndrome: a systematic review and meta-analysis. Sleep Med Rev.

[26] Spiegel K, Knutson K, Leproult R, Tasali E, Van Cauter E (2005). Sleep loss: a novel risk factor for insulin resistance and Type 2 diabetes. J Appl Physiol.

[27] Kim CE, Shin S, Lee H, Lim J, Lee J, Shin A, Kang D (2018). Association between sleep duration and metabolic syndrome: a cross-sectional study. BMC Public Health.

[28] Liu TZ, Xu C, Rota M, Cai H, Zhang C, Shi MJ, Yuan RX, Weng H, Meng XY, Kwong JS (2017). Sleep duration and risk of all-cause mortality: a flexible, non-linear, meta-regression of 40 prospective cohort studies. Sleep Med Rev.

